# Prevalence and Vulnerability Factors Associated with HIV and Syphilis in Older People from Subnormal Agglomerate, Brazilian Amazon

**DOI:** 10.3390/tropicalmed7110332

**Published:** 2022-10-28

**Authors:** Wanderson Santiago de Azevedo Junior, Eduarda Pastana dos Santos, Nábia Pereira Pedreira, Lucas Bittencourt Dantas, Valéria Gabrielle Caldas Nascimento, Geyse Aline Rodrigues Dias, Fabiane de Jesus Dias Sousa, Nádile Juliane Costa de Castro, Eliã Pinheiro Botelho, Glenda Roberta Oliveira Naiff Ferreira

**Affiliations:** 1 Program of Post-Graduation in Nursing, Federal University of Pará, Belém 66075-110, Brazil; 2 Nursing School, Federal University of Pará, Belém 66075-110, Brazil

**Keywords:** HIV, syphilis, mass screening, aged, prevalence

## Abstract

Background: This study aimed to estimate the prevalence of HIV and syphilis and associated factors among elderly people from subnormal agglomerations in a city in the Brazilian Amazon. Methods: An observational, cross-sectional study was carried out in a subnormal agglomerate from the Brazilian Amazon. Data collection was conducted from August 2021 to February 2022, using a structured questionnaire. Whole blood samples were collected to perform a rapid test for HIV and syphilis. People aged 50 and over were included in the study, and the sample consisted of 213 participants. The odds ratio was calculated by multiple logistic regression. Results: A total of 203 participants with a mean age of 63.5 years were considered (95% CI: 62.4; 64.6; standard deviation: 8.1; minimum age: 50 years and maximum age: 94 years). The prevalence of either HIV or syphilis was 16.4% (35/213; 95% CI: 0.11; 0.21); syphilis was 15.5% (33/213) and HIV was 1.40% (3/213). One coinfection (0.46%; 1/213) was registered. In the final multiple logistic regression, the elderly with an education level of illiterate/elementary were two times more likely to have a positive rapid test result for HIV or syphilis. Conclusions: Testing for HIV and syphilis identified that STI represented a burden on populations affected by socioeconomic inequality.

## 1. Introduction

The Human Immunodeficiency Virus (HIV) epidemic and Sexually Transmitted Infections (STIs) represent concerns in the aging population [1,2,3,4]. Contributing to this scenario is the progress of public policies to combat HIV [5] and STIs [6], and the increase in the number of elderly people who remain sexually active [7]. Among STIs, syphilis implies a greater risk of acquiring and transmitting HIV, which can often coexist. Thus, co-infection should be investigated and testing for one should automatically include the other [8,9].

In 2019, among adults over the age of 50 years, 110,000 and 5471 new cases of HIV were diagnosed in the world and in Brazil, respectively [3,10]. While, in England, there were only 850 new HIV diagnoses [2]. Data show that the HIV epidemic disproportionately affects people from low and middle-income countries. The same has been evidenced for syphilis [2,3,10,11]. In South China, there were 34,699 new cases of syphilis, which represented 48.8% of the total [12], while there were 28,130 new cases in Brazil (18% of notified cases) [11]. A lower number were reported in England; 2169 cases in people aged 45 and over [2].

In 2019, in the stratification of different regions in Brazil and among all ages, there were 4948 (11.8% of the total) and 10,621 (6.9% of the total) reported cases of HIV and syphilis in the North Region, respectively [13,14]. This may have been related to the low coverage of primary health care and the system’s capacity to perform population screening [15,16,17,18]. Among people aged 50 and over, an ecological study found 2639 cases of HIV/AIDS reported in Pará between 2007 to 2018, with an annually increasing trend in the HIV detection rate of 30% [18]. In the Brazilian Amazon, this was disproportionately affected by low coverage of primary care services and other social facilities, including schooling, employment, work, and income [15,16,17,18,19].

Epidemiologically, these features may have contributed to the increasing trend of HIV and syphilis observed in the region [10,11,18]. Despite advances in public policies regarding HIV and STIs in Brazil, access to screening, treatment, and treatment monitoring still present key issues, reflected in the high number of AIDS mortality cases. For both infections, the least educated people situated in key populations have been the most affected [3,10,11,13,14,15]. In this region, access to education, culture, and health services, especially HIV and STI policy actions, could reduce exposure to HIV and STI in vulnerable populations [7,18].

In this scenario, screening for HIV and syphilis is essential. Moreover, older people have exhibited low knowledge about STIs [20] and healthcare systems are not prepared to meet the needs of the growing population of older adults at risk of or with an STI [15,21]. Although previous studies have reported prevalence and factors associated with HIV and syphilis, as well as with co-infection, there is a gap in the primary studies of these infections in older people [22,23,24,25], including in the Brazilian Amazon [25]. Studies have compared age groups, but are not focused on older people [2,23,24]. This is perhaps because priority has been given to young people and key populations [3]. Previous studies have reported on factors associated with HIV among those over the age of 50 years: sexual relations with an HIV-infected partner; chemsex; having an HIV-infected partner [7]. Older people have been determined to have greater odds of having active syphilis than those aged 15–24 years [26].

STI screening in older populations is necessary to direct care at the local level, being an essential action of the 90-90-90 strategy [3,5,6]. Additionally, the COVD-19 pandemic has impacted access to screenings for STIs, such as HIV and syphilis, and care-related services. This study aimed to estimate the prevalence of HIV and syphilis and associated factors among elderly people from subnormal agglomerations in a city in the Brazilian Amazon.

## 2. Materials and Methods

### 2.1. Study Design

This observational cross-sectional study was carried out in Guamá sanitary district, composed of six neighborhoods (Canudos, Montese, Guamá, Jurunas, Condor, and Cremação), with 56,669 elderly residents. The Guamá sanitary district is localized in the city of Belém, the capital of Pará state, northern Brazil. Belém has the sixth lowest Municipal Human Development Index (0.746) among the state capitals of Brazil, and only 303,600 inhabitants (20.43%) are covered by the Family Heath Strategy [19].

The site of the study was subnormal agglomerates in Montese, Guamá, and Condor, and were selected due to the local health system in Belém being inconsistently aligned to the health needs of the three neighborhoods and their populations. The neighborhoods are poorly covered by the primary healthcare and populations are affected by social inequality, such as low income, education level, an irregular urban pattern, lack of essential public services, and housing located in areas that are deemed inappropriate for occupation [19]. The sociodemographic and health access context in the Brazilian Amazon requires the adoption of the concept of vulnerability, which implies that the chances of people being exposed to illness depend on social, programmatic, and individual factors [27].

### 2.2. Participants

Data collection was conducted from August 2021 to February 2022. The target population consisted of residents of the Montese, Guamá, and Condor neighborhoods. People aged 50 years and over were included, and those who answered the questionnaire and agreed to undergo both rapid tests for syphilis and HIV were included. Those who agreed to perform only one of the tests and who were unable to answer the questionnaire were excluded. According to the Joint United Nations Programme on HIV and AIDS, every person living with HIV aged 50 and over is considered elderly [28].

### 2.3. Study Size

The sample size was calculated using StatCalc for population survey by Epi Info™ software version 7.2.2.16 (developed by the Centers for Diseases Control and Prevention in Atlanta, GA, USA), and according to the following parameters: a population size of 33,784 elderly (50 years or older) individuals living in the area of study; an acceptable margin of error of five percentage points; a confidence interval of 95%; a design effect of 1.0; an expected prevalence of HIV of 11.7% based on a previous study [7] and an expected prevalence of syphilis of 14.4% based on official data from the Ministry of Health, Brazil [14]. The sample consisted of 188 elderly individuals (calculated by prevalence of syphilis) and included 13% more than the projected sample, resulting in a final sample of 213 enrolled participants.

Considering that there was low coverage of the family health strategy in the study area and there was no list of people aged 50 or over, the sampling technique was based on time location. In this way, the researchers looked for places accessible to and frequented by the elderly in the studied neighborhoods; the selection of collection sites was based both on feasibility of restrictions and on epidemiological criteria. The researchers previously identified the places most frequented by the elderly and with the highest concentration of visitations. Civil society representatives from the neighborhoods were contacted for community mobilization. A schedule with the dates and duration of the collections was prepared. Seven (07) collection actions were carried out in a minimum of six (06) collection hours in each action. The defined locations were parish centers on the main streets, as well as community centers in places with a high flow of elderly people. Before the collections, an advertising campaign was carried out on community radio, in churches, and from bicycles with loudspeakers. For mobilization, a convenience sample was used, due to the lack of a list of patients for random sampling.

### 2.4. Data Sources

The structured questionnaire (Supplementary File) was based on previous studies to assess aspects of vulnerability that interacted with each other to increase or mitigate the chances of exposure to the STIs studied [7,27]. The framework of the concept of vulnerability was used to choose questions (variables) in order to assess access to material and human resources related to HIV/AIDS policies, access to health services, prevention strategies, social aspects and behaviors, attitudes, knowledge, and marital relationships [27].

These relevant aspects were grouped into three vulnerability dimensions: social, programmatic, and individual. The programmatic dimension consists of a focus on policies, programs, and services that may interfere with social and individual aspects. Thus, programmatic aspects can reduce the effects of social inequities and individual behavior [7,27]. The variables were: Did you take a rapid test for HIV? Did you take a rapid test for syphilis? Did you receive condoms at the basic health unit near your home? Did you receive advice from professionals at the basic health unit on transmitting transmitted/prevention of sexually transmitted infections? Did you receive information that you can perform rapid tests at a basic health unit? Did you receive information that you can get condoms at a basic health unit? Did you receive a condom at a basic health unit?

The social dimension refers to cultural, moral, political, economic, and institutional factors; a collective context [27]. The variables grouped into this dimension were: marital status, education level, skin color, and neighborhood.

Finally, the individual dimension is directly influenced by the previous two. This refers to the physical, mental, or behavioral factors that may expose an individual to sexually transmitted infections [7,27]. The variables grouped in this dimension were: gender, sexual orientation, and age (years); Have you had a sexual partner in the last 6 months? Do you use condoms during sex? Do you know how to use condoms? Do you have sex after using alcohol or other legal drugs, age (years).

A draft instrument was sent to researchers to check the content for clarity and adequacy. After these revisions, there were further questionnaire modifications, but no tests were performed to assess the reliability and validity of the questionnaire.

Data were collected by trained researchers through face-to-face interviews. Those who accepted were informed of the objectives and data collected. Privacy was ensured before and after counseling and during testing. Participants were invited to collect blood from the finger pulp of the hand for screening tests for HIV and syphilis.

In whole blood samples, the rapid immunochromatographic test was used for the qualitative determination of total antibodies (IgG, IgM and IgA) against-Treponema pallidum (Kit Syphilis Bio—Bioclin, Minas Gerais, Brazil); no confirmatory testing was used.

HIV screening followed the Brazilian protocol; the rapid immunochromatographic test was used for the qualitative determination of total antibodies in whole blood samples (IgG and IgM) against-human immunodeficiency virus (Kit ABON HIV 1/2/O Tri-line—Abon Biopharm—Hangzhou, China). In case of reagent results, another test was performed (DPP HIV-1/2 from Bio-Manguinhos—Fundação Oswaldo Cruz, Rio de Janeiro, Brazil) in sequence. There was no disagreement between the results. Testing followed manufacturer guidelines.

In post-counseling, a trained nurse delivered the results to the participants. Non reagent tests were considered negative. Those with reactive results for syphilis were referred to the nearest basic health unit, and participants with reactive results for HIV were referred to the municipality’s Testing and Counseling Center.

### 2.5. Variables

The main hypothesis of the study was that factors of social, individual, and programmatic vulnerability could predict the studied STIs (HIV/syphilis).

Dependent variable: to test this hypothesis, the dependent variable was a rapid test result. This variable was treated as dichotomous (reagent and non-reagent). The response event selected was ‘reagent result’. The individual outcomes analyzed were: (1) result of HIV or syphilis or co-infection; (2) syphilis result only; (3) HIV result only.

The independent variables were the dimensions of individual, social, and programmatic vulnerability.

The age variable (years) was analyzed as a quantitative variable and described as mean and standard deviation. The D-Agostino normality test was applied.

### 2.6. Bias

The recall bias was restricted to some questions regarding access to health services and the participants had the same social, economic, and health service access characteristics. Variables with a percentage of non-response above 15% were excluded to avoid bias.

### 2.7. Statistical Analysis

A database was created using the EPI INFO software version 7.2.3 (developed by the Centers for Diseases Control and Prevention in Atlanta, GA, USA). Categorical variables were expressed as percentages and were calculated by the total of the line. The unanswered questions (missing data) did not have their percentage calculated and were not included in the statistical analysis.

Estimates of the prevalence of infection and its confidence interval were calculated by estimating the proportion in the Bioestat software version 5.3^®^ (developed by Mamirauá Institute for Sustainable Development, Belem, Para, Brazil). 

The hypothesis ‘vulnerability factors could predict HIV infection’ was tested by Fischer’s exact test, due to low HIV prevalence.

For other analyses: First, a bivariate analysis compared infected and non-infected participants on vulnerability dimensions. Second, to identify factors independently related to infection, all factors with a level of significance less than 0.2 were included in a multiple logistic regression. Lastly, only variables with *p* < 0.05 were considered.

A crude OR and adjusted odds ratio (AOR) with their respective 95% CIs were used to evaluate the effect. All *p* values < 0.05 were considered statistically significant. To interpret the results, 95% CI and OR were considered in the regression.

### 2.8. Ethical Aspects

This study was part of the project: ‘Situational Diagnosis of Sexually Transmitted Infections in the Amazon Context: Geospatial Analysis, Screening, and Development of Educational Care Technologies’. It was approved by the Research Ethics Committee of the Federal University of Pará, under protocol No. n° 3.331.577. The invited participants who agreed to participate signed an Informed Consent Form, and followed the principles of the Declaration of Helsinki.

## 3. Results

The study sample comprised 213 participants. Among the sociodemographic characteristics, 54.5% (114/209) were illiterate or had elementary education. As for marital status, most participants (59.6%; 121/203) declared as not being in a relationship (single/separated/divorced/widow). As for skin color/ethnicity, most participants (88.1%; 155/211) declared as brown (as classified by the Brazilian Institute of Geography and Statistics). Regarding the neighborhood, 72.7% (155/213) participants lived in the Montese (data shown in Table 1). 

The prevalence of antibodies against HIV or syphilis was 16.4% (35/213; 95% CI: 11.5–21.4%); only syphilis was 15.5% (33/213; 10.6–20.4%) and only HIV was 1.40% (3/213). One coinfection (0.46%; 1/213) was registered among the total number of participants. Among participants who tested positive for syphilis, 1 in 33 tested positive for HIV (3.03%; 1/33).

Table 1 shows that there was no association between social vulnerability factors and HV or syphilis.

The programmatic factors and the results of the HIV or syphilis are shown in Table 2. There was no association between programmatic vulnerability factors and HIV infection or syphilis.

There was a small predominance of women, 61% (130/213), and the majority were heterosexual (97.1%; 203/209). Mean age was 63.5 years (minimum age: 50 years and maximum age: 94 years) (data shown in Table 3).

Table 3 shows the individual factors associated with the presence of antibodies against HIV or syphilis among people aged 50 and over. People aged 50 and over who were bisexual/homosexual were five times more likely to have a reagent test for STI HIV or syphilis (OR = 5.34; 95% CI: 1.03; 27.6; *p* = 0.04), and only syphilis (OR: 5.7; 95% CI: 1.1; 29.9; *p* = 0.03).

For the multiple logistic regression (*p* < 0.20) of HIV or syphilis, the selected variables were: ‘Education level’, ‘Did you take a rapid test for syphilis’, and ‘Sexual orientation’. The results showed that the elderly with an education level of illiterate/elementary were two times more likely to have a positive rapid test result for HIV or syphilis. The results showed that the elderly with an education level of illiterate/elementary were two times more likely to have a positive rapid test result for HIV or syphilis (Table 4).

For the multiple regression of syphilis (*p* < 0.20), the selected variables were: ‘Education level’, ‘Marital status’, ‘Sexual orientation’, and ‘Sex after using alcohol or other legal drugs’. In the final multiple logistic regression model, there was no association between vulnerability factors and syphilis (Table 4).

## 4. Discussion

This cross-sectional study determined the factors associated with HIV and syphilis among people over the age of 50 years living in a subnormal agglomerate in a city in the Brazilian Amazon. Among the 203 participants, the prevalence of HIV or syphilis was 16.4%; only syphilis was 15.5% and only HIV was 1.40%. The prevalence of HIV–syphilis co-infection was 0.46% among the total number of participants, and 3.03% of HIV and syphilis among participants who tested positive for syphilis. The results showed that only one social factor was associated with HIV or syphilis. In the final model, the elderly with an education level of illiterate/elementary were two times more likely to have a positive rapid test result for HIV/syphilis.

For the elderly population, age alone is not a condition identified by the priority testing strategies of public health policies. However, due to the aging of the world’s population, the 2020–2030 decade has been established as the decade of healthy aging. In this population, knowledge of serological status and treatment for HIV and syphilis is critical due to the impacts related to treatment for HIV and age-related diseases [4,5,21]. In recent decades, several studies on the prevalence of HIV and syphilis have been conducted with different populations in the Amazon region, but none specifically with elderly people [25].

The prevalence of syphilis detected in the present study was similar to the 16.1% (55/342) found among people aged 48 years and over in four cities of Marajo Island, Pará, Brazilian Amazon [29].

However, higher seroprevalence has been detected among men who had sex with men in Shenzhen, China, from 2009 to 2017. The prevalence of HIV was 15.26% (38/249); syphilis was 27.71% (69/249); and HIV/syphilis co-infection was 9.24% (23/249) in aged > 50 years [24]. Among people living with HIV, the prevalence of syphilis (co-infection) was 2.1% (11/511) among those with aged over 50 years in the African [23] and 3.7% (10/270) in people aged over 40 years with HIV in Belem, Brazilian Amazon [30]. In Zimbabwe, among 222 cases of syphilis, 45% (100) were with HIV, the only factor associated with syphilis in both females and males at *p* < 0.001 [26].

The incidence of HIV is higher among people with syphilis infection. The syphilis infection almost triples the risk of HIV acquisition [8]. Several studies have been conducted with people living with HIV, increasing the prevalence of syphilis [8,24,30]. In England, a trend over age and time showed that rates of STI were increasing in the older age groups, including those aged 45–64 years. Between 2014 and 2019, there were 5336 new HIV diagnoses in this group; 72% (n = 3834) of which were in men and with significant decreasing trend for late diagnosis in older people [2].

The current study found that in a univariate analysis, bisexual/homosexual individuals are the most vulnerable group for the STIs studied. However, in the multiple regression, only elderly people with a low level of education were associated with HIV or syphilis. The study site is characterized by irregular occupations in an urban area, with little provision of public services, including primary health care and education [19]. In a neighborhood of this subnormal agglomerate, people aged 50 and over are more likely to have low knowledge regarding STIs [31]. It is important to know the factors relevant to different populations, not only in terms of age group and sexual orientation, but also to know the scenarios in which they live, in order to propose intersectoral interventions and, in the health sector, meet the needs of the population [24,25,26,27,29,30,31].

Since 2012, the Brazilian Health Ministry has decentralized rapid testing to primary healthcare [32]. Brazil has one of the world’s largest universal health systems, but access to STI testing can be difficult due to the low coverage of primary care [33]. Therefore, a reverse testing algorithm for syphilis diagnosis is recommended [32], which facilitates testing for HIV and syphilis in populations outside basic health units, especially in the context of a pandemic.

Rapid testing for STI detection in primary health care has increased the number of cases detected and can reduce late diagnoses, promoting timely treatment to reduce adverse STI-related outcomes. In addition, the availability of STI tests makes it possible to identify changes in the demographics of the HIV epidemic, syphilis, and co-infection [2,8]. In England, between 2014 and 2019, there was an increase in costs for HIV care. This was driven by the over 50 years age group, whose costs increased, whereas in younger age groups, costs decreased [2].

Currently, in addition to the growing trend in the increase in the number of cases of elderly people with HIV, syphilis, and other STIs, there is also the aging of the HIV epidemic [2,8,18,23,24,25,26,27,28,29,30,31,32,33,34]. In the United States, fifty percent of people living with HIV in the are now older than 50 years of age. The introduction of antiretroviral therapy, monitoring of viral load and CD4+ cell counts, and better health care regarding the comorbidities that affect the elderly are some factors that contribute to the increase in life quality and life expectancy [34,35].

A cross-sectional study does not make it possible to identify the moment of exposure to an STI. As far as we are aware, this is the first study regarding the screening of HIV and syphilis in people over the age of 50 years of age in the Brazilian Amazon. The generalization of results should consider age, the local health and social context, and similar demographic trajectories. Thus, the results cannot be generalized to the general population. The sample was calculated for a single health district, but was more populous in a municipality with a high incidence of STIs in adults, congenital syphilis, and AIDS mortality. The low number of associated variables found in the study may be related to this small sample, restricted to one district. It is necessary to carry out studies with a larger sample size. A key limitation was the COVID-19 pandemic scenario, due to the fears of the elderly regarding the transmission of COVID-19, limiting the collections to wide spaces in order to maintain the distance between the participants. In addition, the lockdown in January 2022 reduced collections in the Condor neighborhood. Convenience sampling was minimized by mapping the most frequented places and the highest flow of elderly people, including the times of day. In these neighborhoods, entertainment spaces for the elderly are rare. Some questions depended on the participants’ memory, but to reduce this bias, we sought to reduce the number of questions with ‘how long’ or ‘what is the number/value’.

## 5. Conclusions

The prevalence of HIV or syphilis among the elderly was 16.4%, with a lower prevalence of HIV compared to syphilis. Co-infection was detected but had a low frequency among the elderly studied. The factor associated with the presence of markers of HIV infection or syphilis was the low level of education.

Testing actions must be articulated in different spaces outside the health service environment, and it is important that health education actions are directed to the needs of this population and that the care provided to the elderly with STIs considers the impact of HIV and syphilis in the aging process.

This study contributed to the knowledge of the prevalence of HIV and syphilis, and analyzed how programmatic and social variables interact to mitigate the effects of behavior, attitudes, and knowledge that make up the individual dimension.

## Figures and Tables

**Table 1:** Social factors associated with sexually transmitted infections in the elderly in a subnormal agglomeration in the Brazilian Amazon. 2021–2022.

Social	Total	HIV+ or Syphilis+ *		Syphilis *		HIV **	
	n (%)	n (%)	*p* (OR; 95% CI)	n (%)	*p* (OR; 95% CI)	n (%)	*p*
Education level							
Illiterate/Elementary	114 (54.5)	23 (20.2)	0.06 (2.1; 0.9–4.7)	22 (19.3)	0.05 (2.3; 1.0–5.2)	2 (1.8)	1.0
High school/Higher education	95 (45.5)	10 (10.5)	Ref.	9 (9.5)	Ref.	1 (1.1)	
NI	4	2		2			
Marital status							
Single/Separated/ divorced/widow(er)	121 (59.6)	22 (18.2)	0.3 (1.4; 0.6–3.1)	22 (18.2)	0.1 (1.8; 0.8–4.1)	0	0.16
Married/stable union	82 (40.4)	11 (13.4)	Ref.	9 (11)	Ref.	2 (2.4)	
NI	10	2		2		1	
Skin colour							
Black	35 (16.6)	7 (20.0)	0.7 (0.8; 0.2–2.9)	7 (20)	0.9 (1.06; 0.3–4.1)	0	0.3
Brown	155 (73.5)	23 (14.8)	0.3 (0.5; 0.2–1.6)	22 (14.2)	0.5 (0.7; 0.2–2.3)	2 (1.3)	0.3
Yellow/white	21 (10)	5 (23.8)	Ref.	4 (19)	Ref.	1 (4.8)	
NI	2	0					
Neighbourhood							
Montese	155 (72.8)	26 (16.8)	0.7 (1.2; 0.4–2.9)	24 (15.5)	0.8 (1.1; 0.4–2.7)	2 (1.3)	0.9
Condor	10 (4.7)	2 (20)	0.6 (1.4; 0.2–8.3)	2 (20)	0.6 (1.4; 0.2–8.3)	0	1.0
Guamá	48 (22.5)	7 (14.6)	Ref.	7 (14.6)	Ref.	1 (2.1)	

Legend: * Binary regression. ** Fischer’s exact test. NI: not informed—Not considered for statistical calculation. Ref.: reference. CI: confidence intervals. OR: odds ratio.

**Table 2:** Programmatic factors associated with sexually transmitted infections in older people in a subnormal agglomeration in the Brazilian Amazon. 2021–2022.

Programmatic	Total	HIV+ or Syphilis+ *		Syphilis *		HIV **	
	n (%)	n (%)	*p* (OR; 95% CI)	n (%)	*p* (OR; 95% CI)	n (%)	*p*
Did you take a rapid test for HIV							
Yes	93 (44.3)	14 (15.1)	Ref.	12 (12.9)	Ref.	2 (2.2)	0.58
No	117 (55.7)	21 (17.9)	0.6 (1.2; 0.6–2.6)	21 (17.9)	0.3 (1.4; 0.7–3.1)	1 (0.9)	
Don’t remember	3	0					
Did you take a rapid test for syphilis							
Yes	79 (38.9)	17 (21.5)	Ref.	1519)	Ref.	3 (3.8)	0.05
No	124 (61.1)	17 (13.7)	0.1 (0.6; 0.2–1.2)	17 (13.7)	0.3 (0.7; 0.3–1.4)	0	
NI/don’t remember	10	1		1			
Received condoms at the basic health unit close to home							
Yes	58 (28.3)	11 (19)	Ref.	11 (19)	Ref.	0	0.56
No	147 (71.7)	22 (15)	0.5 (0.7; 0.3–1.6)	20 (13.6)	0.3 (0.7; 0.3–1.5)	3 (2)	
NI	8	2		2			
Received advice from professionals at the basic health unit on transmitting/prevention of sexually transmitted infections							
Yes	111 (53.9)	16 (14.4)	Ref.	14 (12.6)	Ref.	2 (1.8)	1.0
No	95 (46.1)	18 (18.9)	0.4 (1.4; 0.6–2.9)	18 (18.9)	0.2 (1.6; 0.7–3.4)	1 (1.1)	
NI	7	1		1			
Received information that you can perform rapid tests on basic health unit							
Yes	94 (45.4)	12 (12.8)	Ref.	12 (12.8)	Ref.	0	0.5
No	113 (54.6)	21 (18.6)	0.2 (1.5; 0.7–3.3)	20 (17.7)	0.3 (1.4; 0.7–3.1)	2 (1.8)	
NI	6	2		1		1	
Received information that you can get condoms at a basic health unit							
Yes	136 (67.0)	23 (16.9)	Ref.	21 (15.4)	Ref.	3 (2.2)	0.5
No	67 (33.0)	11 (16.4)	0.9 (0.9; 0.4; 2.1)	11 (16.4)	0.8 (1.1; 0.5–2.4)	0	
NI	10	1		1			
Received a condom at a basic health unit							
Yes	54 (26.4)	9 (16.7)	Ref.	9 (16.7)	Ref.		0.5
No	151 (73.6)	25 (16.6)	0.9 (0.9; 0.4; 2.2)	23 (15.2)	0.8 (0.9; 0.4–2.1)	3 (2.0)	
NI	8	1		1		0	

Legend: * Binary regression. ** Fischer’s exact test. NI: not informed—Not considered for statistical calculation. Ref.: reference. CI: confidence intervals. OR: odds ratio.

**Table 3:** Individual factors associated with sexually transmitted infections in older people in a subnormal agglomeration in the Brazilian Amazon. 2021–2022.

Individual	Total	HIV+ or Syphilis+ *		Syphilis *		HIV **	
	n (%)	n (%)	*p* (OR; 95% CI)	n (%)	*p* (OR; 95% CI)	n (%)	*p*
Age (years)							
Mean (SD)	63.5 (8.1)	62.4 (8.8)	0.4 (0.9; 0.9–1.0)	62.2 (9.0)	0.3 (0.9; 0.9–1.0)	64.3 (2.5)	
Sexual orientation							
Heterosexual,	203 (97.1)	32 (15.8)	Ref.	30 (14.8)	Ref.	3 (1.5)	1.0
Bisexual/homosexual	6 (2.9)	3 (50)	0.04 (5.3; 1.0–27.6)	3 (50)	0.03 (5.7; 1.1–29.9)	0	
NI	4						
Gender							
Women	130 (61.0)	20 (15.4)	Ref.	19 (14.6)	Ref.	2 (1.5)	1.0
Men	83 (39.0)	15 (18.1)	0.6 (1.2; 0.6–2.5)	14 (16.9)	0.6 (1.2; 0.5–2.5)	1 (1.2)	
Sexual partnership in the last 06 months							
Fixed/Never had sex/None sexual partnership	156 (82.1)	23 (14.7)	Ref.	21 (13.5)	Ref.	2 (1.3)	1.0
Causal sex/more than 01	34 (17.9)	7 (20.6)	0.4 (1.5; 0.6–3.8)	7 (20.6)	0.3 (1.6; 0.6–4.3)	0	
NI	23	5		5		1	
Use of condoms during sexual intercourse							
Always	48 (24.2)	9 (18.8)	Ref.	8 (16.7)	Ref.	2 (4.2)	0.14
No/Sometimes	150 (75.8)	22 (14.7)	0.5 (0.7; 0.3–1.7)	21 (14)	0.6 (0.8; 0.3–2.0)	1 (0.7)	
NI	15	4		4			
Know how to use condom							
Yes	145 (71.8)	23 (15.9)	Ref.	21 (14.5)	Ref.	2 (1.4)	1.0
No	57 (28.2)	9 (15.8)	0.9 (0.99; 0.4–2.3)	9 (15.8)	0.8 (1.1; 0.4–2.6)	0	
NI	11	3		3		1	
Sex after using alcohol or other legal drugs							
No	171 (84.7)	25 (14.6)	Ref.	23 (13.5)	Ref.	2 (1.2)	
Yes	31 (15.3)	7 (22.6)	0.26 (1.7; 0.6–4.3)	7 (22.6)	0.19 (1.9; 0.7–4.8)	0	1.0
NI	11	3		3		1	

Legend: * Binary regression. ** Fischer’s exact test. NI: not informed—Not considered for statistical calculation. Ref.: reference. CI: confidence intervals. OR: odds ratio.

**Table 4:** Results of the multiple logistic regression analysis between factors Sexually Transmitted Infections among elderly in a subnormal agglomeration in the Brazilian Amazon. 2021–2022.

Variables	HIV or Syphilis		Syphilis	
	*p*	AOR (95% CI)	*p*	AOR (95% CI)
Education level (Ref.: High school/Higher education)				
Illiterate/Elementary	0.03	2.50 (1.08; 5.81)	0.07	1.6 (0.6; 4.0)
Marital status (Ref.: Married/stable union)				
Single/Separated/divorced/widow(er)			0.30	2.3 (0.9; 5.8)
Did you take a rapid test for syphilis? (Ref.: Yes)				
No	0.21	0.61 (0.27; 1.33)		
Sexual orientation (Ref.: Heterosexual)				
Bisexual/homosexual	0.09	5.16 (0.74; 35.9)	0.09	5.3 (0.7; 38.3)
Sex after using alcohol or other legal drugs (Ref.: No)				
Yes			0.20	1.9 (0.7; 5.6)

Legend: Adjusted Odds Ratio. CI: confidence intervals. Ref.: reference.

## Data Availability

The original contributions presented in the study are included in the article, further inquiries can be directed to the corresponding author to glendaf@ufpa.br.

## Supplementary Materials

The following supporting information can be downloaded at: https://www.mdpi.com/article/10.3390/tropicalmed7110332/s1, Supplementary file: structure questionnaire.
